# Earthworm-Mycorrhiza Interactions Can Affect the Diversity, Structure and Functioning of Establishing Model Grassland Communities

**DOI:** 10.1371/journal.pone.0029293

**Published:** 2011-12-28

**Authors:** Johann G. Zaller, Florian Heigl, Andrea Grabmaier, Claudia Lichtenegger, Katja Piller, Roza Allabashi, Thomas Frank, Thomas Drapela

**Affiliations:** 1 Department of Integrative Biology and Biodiversity Research, Institute of Zoology, University of Natural Resources and Life Sciences Vienna, Vienna, Austria; 2 Institute of Sanitary Engineering and Water Pollution Control, University of Natural Resources and Life Sciences Vienna, Vienna, Austria; University of Guelph, Canada

## Abstract

Both earthworms and arbuscular mycorrhizal fungi (AMF) are important ecosystem engineers co-occurring in temperate grasslands. However, their combined impacts during grassland establishment are poorly understood and have never been studied. We used large mesocosms to study the effects of different functional groups of earthworms (i.e., vertically burrowing anecics vs. horizontally burrowing endogeics) and a mix of four AMF taxa on the establishment, diversity and productivity of plant communities after a simulated seed rain of 18 grassland species comprising grasses, non-leguminous forbs and legumes. Moreover, effects of earthworms and/or AMF on water infiltration and leaching of ammonium, nitrate and phosphate were determined after a simulated extreme rainfall event (40 l m^−2^). AMF colonisation of all three plant functional groups was altered by earthworms. Seedling emergence and diversity was reduced by anecic earthworms, however only when AMF were present. Plant density was decreased in AMF-free mesocosms when both anecic and endogeic earthworms were active; with AMF also anecics reduced plant density. Plant shoot and root biomass was only affected by earthworms in AMF-free mesocosms: shoot biomass increased due to the activity of either anecics or endogeics; root biomass increased only when anecics were active. Water infiltration increased when earthworms were present in the mesocosms but remained unaffected by AMF. Ammonium leaching was increased only when anecics or a mixed earthworm community was active but was unaffected by AMF; nitrate and phosphate leaching was neither affected by earthworms nor AMF. Ammonium leaching decreased with increasing plant density, nitrate leaching decreased with increasing plant diversity and density. In order to understand the underlying processes of these interactions further investigations possibly under field conditions using more diverse belowground communities are required. Nevertheless, this study demonstrates that belowground-aboveground linkages involving earthworms and AMF are important mediators of the diversity, structure and functioning of plant communities.

## Introduction

In temperate grasslands, earthworms and arbuscular mycorrhizal fungi (AMF) are among the most important heterotrophic soil organisms by making up the dominant fraction of soil fauna [1] or forming symbiotic associations with the majority of land plants [2]. Because of their eminent influence on ecosystem characteristics and functions, both earthworms and AMF are considered as ecosystem engineers in many terrestrial ecosystems [3], [4], [5]. Previous work has shown that both earthworms [6], [7], [8], [9] and AM fungi [10], [11], [12], [13] can individually affect or be affected by grassland plant diversity and nutrient cycling. Nevertheless, despite this unequivocal importance we know very little about their combined effects especially in the phase of grassland establishment.

Earthworm communities in temperate grasslands in Europe usually comprise species belonging to three functional groups, anecics or vertical burrowers, endogeics or horizontal burrowers and epigeics or surface dwellers mainly distinguished because of their behaviour, activity zones and food preferences within the soil [14], [15], [16]. Important mechanisms by which earthworms can affect the diversity and structure of plant communities are by (i) selective feeding on plant seeds [8], [17], [18], [19], (ii) bidirectional transport of plant seeds in the soil seed bank [8], [20], (iii) the deposition of nutrient-rich earthworm casts near specific plant species and thereby favouring their growth [21], (iv) seedling recruitment [22] or (v) altering plant competition and production [23], [24], [25], [26]. In temperate grasslands despite seeds present in the soil seed bank, a great amount of seedling recruitment occurs via seed rain which can amount to almost 11000 seeds m^2^ year^−1^ [27]. It can be expected that the germination of these seeds will be affected if earthworms feed upon them or remove them from the soil surface. Moreover, through burrowing, casting and mixing of litter and soil, earthworms impact microbial activity and nutrient availability in the soil [28], [29], [30], modifying the soil structure by producing stable macropores and aggregates [31], [32], [33] and influence soil water characteristics by increasing water and nutrient infiltration in soils [34], [35], [36], [37], [38].

Arbuscular-mycorrhizal fungi have also been shown to play key roles (i) as support system for seedling establishment [39], (ii) plant growth [40], [41], (iii) ecosystem nutrient cycling [42], [43], [44] and (iv) nutrient leaching from ecosystems [45], [46].

We already know that earthworms and AMF interact directly as earthworms were shown to selectively feed on fungal mycelia [47], disperse AMF spores [48], [49], [50], increase AMF biomass in the soil [51] and either increase [52] or do not affect root AMF colonisation [53]. Consequences of earthworm-AMF interactions on plant performance are commonly species-specific and vary from an increased plant nutrient uptake and productivity [54], [55], [56] to no interactive effects [53], [57].

For the current study we hypothesized that anecic and endogeic earthworms will specifically interact with AMF and will due to their different burrowing and feeding behaviour differently affect plant parameters. Specifically we hypothesized that: (i) Aboveground, anecic earthworms due to selective seed removal and/or consumption will alter the establishment of seeds landing on the soil surface more than endogeics. (ii) Belowground, both groups of earthworms will alleviate AMF colonization of seedlings by stimulated root growth and/or AMF transport. (iii) Earthworms and AMF will both increase plant production by increasing nutrient availability. (iv) By their burrowing, anecic earthworms will increase water infiltration and nutrient leaching out of the system more than endogeic earthworms; AMF is expected to buffer this effect by stimulating growth, and water and nutrient uptake of plants.

These hypotheses were tested in a full-factorial experiment using large mesocosms where single and combined effects of two earthworm species and four AMF taxa on the establishment of 18 plant species entering the system via a simulated seed rain were studied. As climate models predict more frequent extreme events such as heavy rainfalls until the end of the 21^st^ century [58] we also tested whether earthworm-AMF interactions affect important ecosystem services such as water infiltration and nutrient leaching after a simulated heavy shower.

## Materials and Methods

### Study system

The experiment was conducted between April and July 2009 in a greenhouse at the University of Natural Resources and Life Sciences Vienna (BOKU), Austria. We used 20 l plastic pots (diameter: 31 cm, height: 30 cm; further called mesocosms) filled with steam-sterilized (3 hours at 100°C) field soil (Haplic Chernozem, silty loam) mixed with quartz sand (grain size 1.4–2.2 mm) in a ratio of 40∶60 vol/vol (nutrient contents of the soil mixture: C_org_ = 24.1 g kg^−1^, N_tot_ = 0.98±0.09 g kg^−1^, K = 111.2±0.8 mg kg^−1^, P = 58.42±0.53 mg kg^−1^, pH = 7.63±0.03). We successfully used this substrate mixture in other experiments involving the same plant, earthworm and AMF taxa [41], [56], [59], [60]. Before filled with soil, mesocosms were lined out with two layers of planting fleece at the bottom and extended at the upper rim with a 20-cm high barrier of transparent plastic to prevent earthworms from escaping; the fleece and barriers were also installed in mesocosms containing no earthworms to create similar microclimatic conditions among treatments. Mesocosms were randomly placed on the greenhouse floor. Mean daily air temperature during the course of the experiment was 21.9°C at a mean relative humidity of 56.7%.

### Experimental Setup

To establish the treatment AMF+, mesocosms were first filled with 6 l steam-sterilized field soil/quartz sand mixture (making a 10 cm thick layer at the bottom of the mesocosms) amended with 37.5 g of inoculum of *Glomus intraradices* (N.C. Schenck & G.S. Sm.), *G. claroideum* (N.C. Schenck & G.S. Sm.), *G. mosseae* (T.H. Nicolson & Gerd.) and *G. geosporum* (T.H. Nicolson & Gerd.) obtained from a commercial supplier (Symbio-m Ltd., Landskroun, Czech Republic). The AMF controls (treatment AMF−) were filled with the same amount of steam-sterilized AMF inoculum. The AMF layer was then covered with 12 l of steam-sterilized soil mixture containing no AMF inoculum until 2 cm below the upper rim (in total 18 l substrate in mesocosms).

Earthworms were added to the mesocosms in the following manner: treatment Ac received four specimens of *Aporrectodea caliginosa* (Savigny 1826) per mesocosm (total earthworm biomass 4.47±1.01 g mesocosm^−1^ - mean ± SE; equivalent to 60 g m^−2^); treatment Lt received two adult specimens of *Lumbricus terrestris* (Linnaeus 1758) per mesocosm (10.33±2.14 g mesocosm^−1^; equivalent to 138 g m^−2^); treatment AcLt received two *A. caliginosa* and one *L. terrestris* (6.98±1.31 g mesocosm^−1^; equivalent to 93 g m^−2^); treatment NoEw received no earthworms. Earthworm treatments were roughly oriented on the average earthworm biomass in temperate grasslands ranging between 52–305 g m^−2^, where 50–75% of the biomass consists of anecic species [1]. Although earthworm communities in temperate grasslands also comprise surface dwelling epigeic species [6], [16], we did not include epigeics in this experiment because they (i) make up a much lower biomass contribution than anecics and endogeics, (ii) would presumably consume the majority of seeds from the soil surface making comparisons between all three groups impossible and (iii) are not thought to interact with AMF present in the soil. When arranging the earthworm treatments we deliberately did not want to create treatments with similar earthworm biomass as the burrowing activity of species usually differ considerably thus giving wrong impressions of their impact (i.e., the smaller endogeic *A. caliginosa* is commonly more active than the bigger *L. terrestris*; [61]) and decided for lower endogeic biomass than anecic biomass in our treatments. We collected *A. caliginosa* in a garden soil near the city of Eisenstadt (Burgenland) by hand digging. The garden is owned by an author of this study (JGZ) who gave permission to collect earthworms therein. *Lumbricus terrestris* was obtained from a fishing bait shop in Vienna. To ensure that earthworms do not carry over AMF from field soil we cultivated them in sterile soil in a dark climate chamber (15°C) for one week and relocated them into new sterile substrate for another four days; during this quarantine, earthworms were regularly fed with ground oat flakes. After eleven days in the climate chamber, earthworms were carefully washed free of soil, placed on moist filter paper, weighed and inserted into mesocosms. The majority of earthworms buried themselves in the soil within a few minutes; earthworms that were still on the surface the next day were replaced by new specimens cultivated in sterile substrate in the climate chamber. Although, *A. caliginosa* is considered a soil dwelling species it can frequently be observed on the soil surface, especially during rainy weather.

One day after earthworm insertion, a seed rain was simulated by randomly spreading eight seeds of each of the below-mentioned 18 grassland species on the soil surface (totally 1900 seeds m^−2^). We used seeds of seven grass species (*Arrhenatherum elatius* L., *Brachypodium pinnatum* L., *Bromus erectus* Huds., *Cynosurus cristatus* L., *Dactylis glomerata* L., *Festuca ovina* L., *Holcus lanatus* L.); seven non-leguminous forb species (further called forbs; *Centaurea jacea* L., *Hieracium pilosella* L., *Knautia arvensis* L., *Leontodon hispidus* L., *Leucanthemum ircutianum* Mill., *Plantago lanceolata* L., *Salvia pratensis* L.) and four leguminous forbs (further called legumes; *Anthyllis vulneraria* L., *Lotus corniculatus* L., *Trifolium pratense* L., *Vicia cracca* L.). Seed material was obtained from a commercial supplier who guaranteed germination rates above 95% (Rieger- Hofmann GmbH, Blaufelden-Raboldshausen, Germany). We chose these plant species because they can frequently be found in low-fertile grasslands in Central Europe [62]. All species used in this experiment are commonly co-occurring in low-fertile Central European grasslands.

These treatments were replicated five times in a full-factorial design: four earthworm treatments (Ac, Lt, AcLt, no earthworms)×two AMF treatments (inoculation of the AMF mix, no AMF inoculation)×five replicates amounting to totally 40 mesocosms. All mesocosms were watered with a constant amount of tap water according to temperature and humidity conditions in the greenhouse; no fertilizers were applied during the course of the experiment.

### Measurements and Analyses

Seedling establishment was counted on average every five days up to 52 days after seeding. Because of difficulties in identifying small seedlings only total number of emerging seedlings were counted.

After 12 weeks, the mesocosms were watered to field capacity and subsequently received 3 l of distilled water simulating a rain shower of about 40 l m^−2^. Time from pouring the water onto the mesocosms until the last water pool disappeared was recorded and used to calculate the water infiltration rate in l m^−2^ s^−1^. The water solution that leached through the soil of each mesocosm was collected in bottles and immediately stored at −20°C until further analyses. Nitrate (NO_3_^−^) was determined by ion-chromatography (ICS 3000; Dionex, Bannockburn, IL, USA), at the Institute of Sanitary Engineering and Water Pollution Control at BOKU, according to standard methods EN ISO 10304-1 (1995) and EN ISO 10304-2 (1996). Ammonium (NH_4_^+^) and phosphate (PO_4_^3−^) concentration were determined by spectrophotometrically (U2001; Hitachi, Tokyo, Japan) at the same lab, according to DIN 38406-1 (1983) and EN ISO 6878 (2004), respectively.

After assessing water infiltration and nutrient leaching, the number of plant individuals (plant density per mesocosm) were counted. Afterwards, plant harvest started by flipping over the mesocosms and searching for earthworms in the soil for seven minutes per mesocosm. Thereafter, each individual plant was carefully excavated, shoots were cut off and roots washed free of attached soil particles under a jet of water in a 1 mm sieve. Dry mass of shoots and roots was determined after drying for 48 hours at 55°C. A portion of roots was collected, stained with ink [63] and the percentage of root length colonised by AMF was determined using the grid-line method by counting at least 100 intersections per sample [64].

### Statistical analysis

We tested all variables for homogeneity of variances and normality using the tests after Levene and Kolmogorow-Smirnow, respectively [65]. Assumptions for parametric tests were fulfilled by all tested parameters. Seedling germination over time was analysed using a repeated measures ANOVA with earthworm treatments and AMF inoculation as factors. Data on plant community diversity, density, water infiltration and nutrient leaching were analysed using two way ANOVAs. Further, separately for mesocosms without and with AMF one-way ANOVAS were conducted to be able to determine effects at the plant functional group level. Each ANOVA was followed by Tukey-HSD post-hoc comparisons with sequential Bonferroni corrections to account for differences between earthworm treatments. We used Pearson correlations to test for the relationship between water infiltration, nutrient leachate, plant community parameters and earthworm biomass. All statistical tests were performed using the GLM procedure in SPSS (vers. 17.0.0, SPSS Inc. Headquarters, Chicago, Illinois, USA). Values given throughout the text are means ± SE.

## Results

### Earthworms and mycorrhization

Earthworm recapture rates based on fresh mass varied significantly between earthworm species (43.7±3.6%, 57.8±4.2% and 75.8±2.4% recapture for the treatments Ac, Lt and AcLt, respectively; F_3,69_ = 4.702, P = 0.005) but did not differ between AMF treatments (AMF−: 52.1±4.8%; AMF+: 62.5±4.7%; F_1,69_ = 1.032, P = 0.314). Earthworm activity as measured by the cumulated number of earthworm burrow openings was 32.0±7.9, 40.8±6.4, 40.5±4.5 channels per mesocosm for the Ac, Lt and AcLt treatments, respectively and was not affected by earthworm treatments (F_2,59_ = 2.193, P = 0.121) or AMF inoculation (F_1,59_ = 0.136, P = 0.714; no EW×AMF interaction).

The percentage root length colonised by AMF (Table 1) varied significantly between plant functional groups (F_2, 159_ = 5.850, P = 0.004) and was significantly affected by earthworm treatments (F_3, 159_ = 3.728, P = 0.013; plant functional group×earthworm interaction: F_6, 159_ = 1.905, P = 0.083). AMF root colonization reached a maximum of 45% for the forb *C. jacea* in the Lt treatment (data not shown); plant roots in AMF− controls were generally not colonised by AM fungi.

**Table 1:** Percent root length colonized by AMF of legumes, grasses and non-leguminous forbs in mescocosms containing different earthworm species (NoEw…no earthworms, Ac…only *A. caliginosa*, Lt…only *L. terrestris*, AcLt…both species).

	Earthworm treatments			
Variable	NoEw	Ac	Lt	AcLt
All plant species	2.3±0.8b	3.4±1.3b	7.6±2.2a	2.6±1.1b
Legumes	7.3±2.9b	5.9±2.3b	16.6±6.1a	3.8±2.2a
Grasses	1.3±0.8b	6.1±3.5a	2.4±1.4b	0.8±0.6b
Forbs	1.1±0.4b	0.0±0.0c	7.0±2.9a	4.3±3.4b

Only data from mesocosms inoculated with AMF are shown. Different letters within each row represent significant differences (P<0.05; Tukey-test with sequential Bonferroni correction) between earthworm treatments. Means ± SE, n = 5.

### Plant community measures

**Figure 1:**
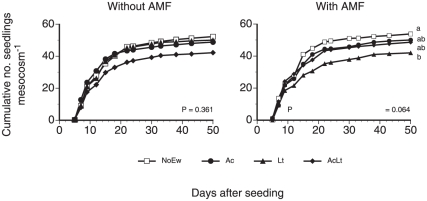
Seedling establishment in mesocosms containing different combinations of earthworm species (NoEw…no earthworms, Ac…*A. caliginosa*, Lt…*L. terrestris*, AcLt…both species) without or with AMF inoculation. P-Values from repeated measurement ANOVAs, different letters denote significant differences at the end of the measurement period (Tukey, P<0.05). Means, n = 5.

Across treatments, from the 144 seeds mesocosm^−1^ placed onto the soil surface, on average 43.8±4.4 seeds mesocosm^−1^ emerged until the end of the experiment (Fig. 1). Repeated measures ANOVAs showed that earthworm treatments tended to affect seedling emergence only in mesocosms containing AMF (F_3,16_ = 2.953, P = 0.064); here mesocosms containing Lt showed less seedlings than mesocosms without earthworms (Fig. 1).

**Figure 2:**
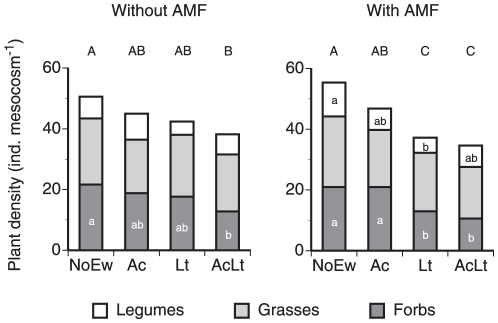
Species diversity of legumes, grasses and forbs in mescocosms containing different earthworm species (NoEw…no earthworms, Ac…*A. caliginosa*, Lt…*L. terrestris*, AcLt…both species) with and without AMF inoculation. Lower case letters denote differences between earthworm treatments within each plant functional group and AMF treatment; upper case letters denote differences between total plant species diversity (P<0.05). Means, n = 5.

Overall diversity of plant communities was only affected by anecic earthworms when AMF were present while endogeics alone or mixed earthworm communities did not affect plant diversity (Fig. 2; Table 2). At the plant functional group level, only legume diversity was significantly affected by earthworms, however remained unaffected by AMF. Earthworm effects on forb diversity varied between AMF treatments (significant earthworm×AMF interaction). Considering only mesocosms without AMF, grass diversity was significantly higher at NoEw than at AcLt (Fig. 2). In mesocosms containing AMF forb diversity in mesocosms with Lt was significantly lower than in Ac (Fig. 2).

**Table 2:** ANOVA results for treatment effects on plant community parameters and microcosm leachate.

	Earthworms		AMF		Ew×AMF	
Variable	F	P	F	P	F	P
**Plant community diversity (# spp.)**	2.725	0.061	0.186	0.669	0.356	0.785
Forb diversity	0.397	0.756	0.19	0.666	3.19	0.038
Grass diversity	2.57	0.073	0.334	0.568	0.486	0.695
Legume diversity	4.405	0.011	0.011	0.918	0.923	0.441
**Plant community density (# ind.)**	4.527	0.009	0.026	0.874	0.461	0.712
Forb density	13.742	<0.001	1.204	0.281	1.451	0.246
Grass density	1.251	0.308	0.003	0.958	0.189	0.903
Legume density	3.781	0.02	0.763	0.389	1.424	0.254
**Total plant biomass (g)**	2.067	0.124	0.055	0.816	0.539	0.659
*Shoot biomass*	2.505	0.077	0	0.988	0.495	0.688
Forb shoot mass	5.741	0.003	3.194	0.083	0.378	0.769
Grass shoot mass	1.691	0.189	3.413	0.074	1.106	0.361
Legume shoot mass	4.766	0.007	2.316	0.138	1.665	0.194
*Root biomass*	1.47	0.241	0.063	0.804	1.407	0.259
Forb root mass	0.964	0.422	0.52	0.477	0.359	0.783
Grass root mass	2.13	0.117	0.007	0.933	2.029	0.131
Legume root mass	0.801	0.502	0.001	0.978	1.03	0.392
**AMF root colonization (%)**	3.224	0.024	n.a.		n.a.	
**Water infiltration (l m^−2^ s^−1^)**	14.103	<0.001	0.065	0.8	2.41	0.074
**Ammonium in leachate (mg l^−1^)**	4.414	0.01	1.039	0.316	0.386	0.763
**Nitrate in leachate (mg l^−1^)**	1.307	0.282	3.606	0.063	0.163	0.921
**Phosphate in leachate (mg l^−1^)**	0.281	0.838	0.073	0.788	0.443	0.724

P-values after sequential Bonferroni corrrections.

**Figure 3:**
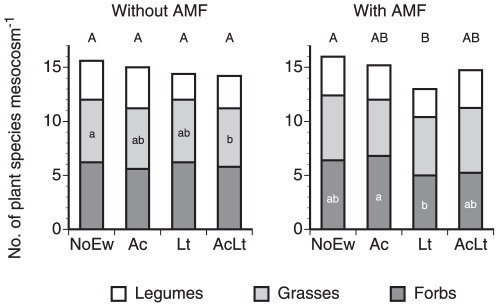
Density of legumes, grasses and forbs in mescocosms containing different earthworm species (NoEw…no earthworms, Ac…*A. caliginosa*, Lt…*L. terrestris*, AcLt…both species) with and without AMF inoculation. Lower case letters represent differences between earthworm treatments within each plant functional group and AMF treatment; upper case letters denote differences between total plant density (P<0.05). Means, n = 5.

Total plant community density was significantly reduced by earthworm treatments but unaffected by AMF treatment (Fig. 3, Table 2). In mesocosms without AMF significantly lower total plant density was observed in AcLt than in NoEw mesocosms mainly because of a reduced forb density. In mesocosms with AMF total plant density was significantly lower in Lt and AcLt than in NoEw mainly because forb density was significantly lower in Lt and AcLt and legume density was lower in Lt than in NoEw. Grass density was unaffected by earthworms or AMF.

**Figure 4:**
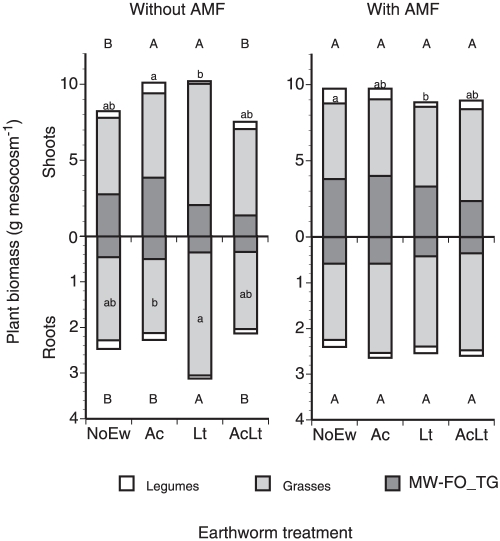
Biomass production of legumes, grasses and forbs in mescocosms containing different earthworm species (NoEw…no earthworms, Ac…*A. caliginosa*, Lt…*L. terrestris*, AcLt…both species) with and without AMF inoculation. Lower case letters denote differences between earthworm treatments within each plant functional group and AMF treatment; upper case letters denote differences between total plant biomass (P<0.05). Means, n = 5.

Total plant biomass production was neither affected by earthworm nor AMF treatments (Fig. 4, Table 2). However, forb and legume shoot mass were significantly affected by earthworms but not affected by AMF. Specific root masses (root length per mass) of functional groups remained unaffected by either earthworms or AMF (data not shown). In mesocosms without AMF, total shoot mass in Ac and Lt was significantly higher than in NoEw or AcLt; of functional groups only legumes in Lt showed significantly lower shoot mass than those in Ac. Total root mass remained unaffected by either earthworms or AMF. When considering mesocosms without AMF only grass root mass under Lt was significantly higher than in Ac. In mesocosms containing AMF neither total shoot mass nor total root was affected, however legume shoot mass was significantly lower in Lt than in NoEw.

### Water infiltration and nutrient leaching

**Figure 5:**
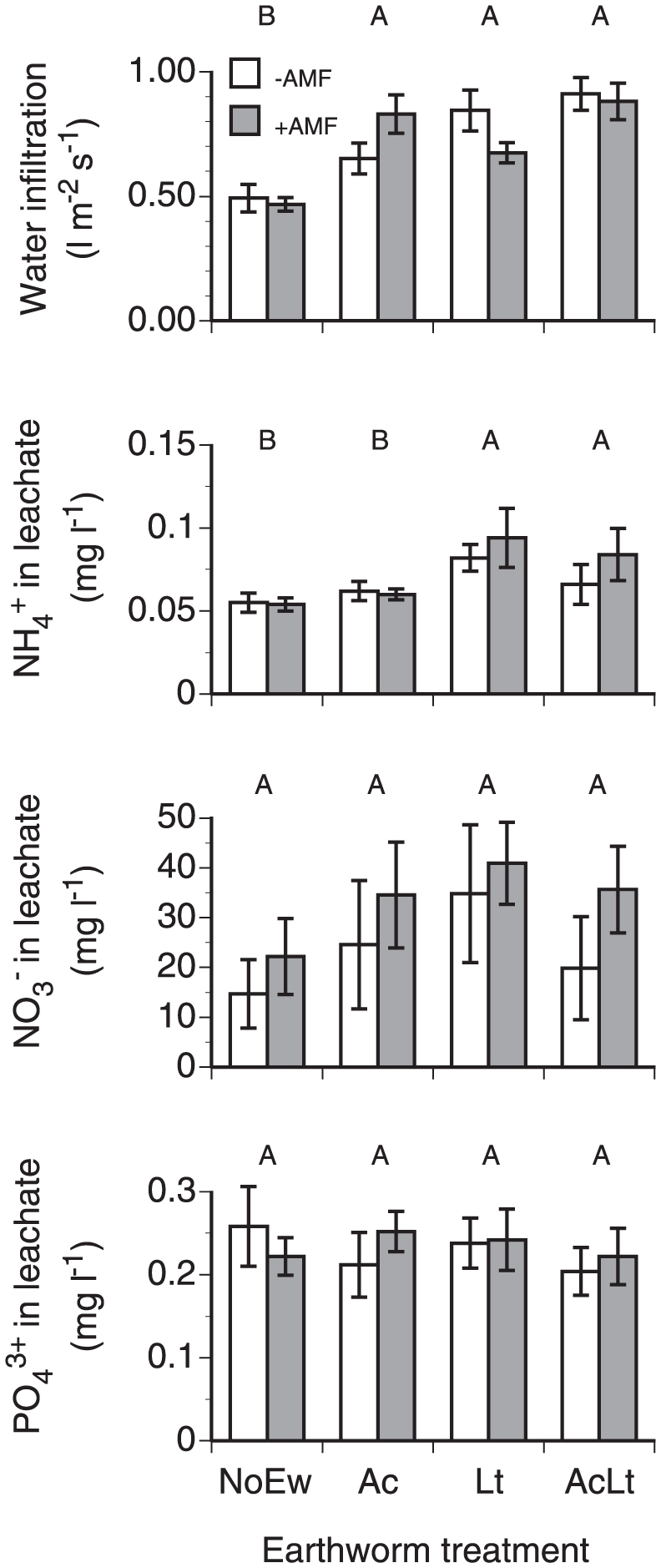
Water infiltration, ammonium, nitrate and phosphate leaching in mescocosms containing different earthworm species (NoEw…no earthworms, Ac…*A. caliginosa*, Lt…*L. terrestris*, AcLt…both species) with and without AMF inoculation. Upper case letters denote differences between earthworm treatments; AMF showed no effects (P<0.05). Means ± SE, n = 5.

Water infiltration rate was significantly higher in mesocosms containing earthworms than in NoEw mesocosms; AMF had no effect on water infiltration (Fig. 5, Table 2). Ammonium leaching was significantly higher in Lt and AcLt than in NoEw and Ac but not affected by AMF. Leachate nitrate and phosphate concentrations were unaffected by earthworm or AMF treatments.

### Correlations between plant measures, water infiltration and nutrient leaching

**Figure 6:**
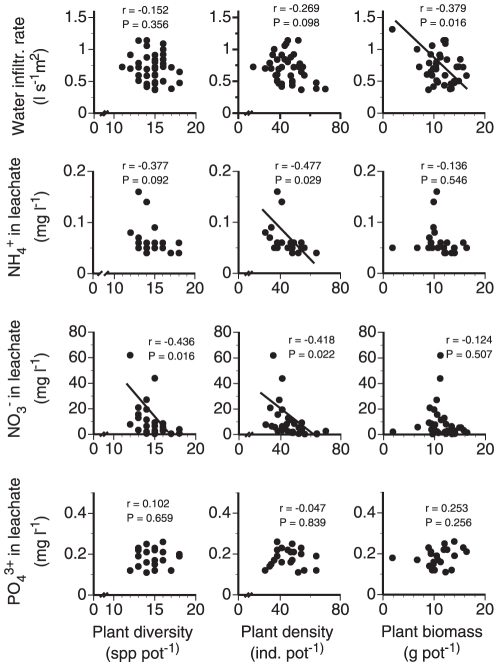
Water infiltration, ammonium, nitrate and phosphate leaching as a function of plant diversity, plant density and total plant biomass in mesocosms containing different earthworm species with and without AMF inoculation. R and P-values from Pearson correlations across all treatments. Means, n = 40 mesocosms.

Water infiltration rate was significantly negatively correlated with mesocosm plant biomass (Fig. 6). Ammonium concentration in leachate was significantly negatively correlated with plant density, nitrate concentration in leachate was highly significantly negative correlated with plant diversity and density. Phosphate concentration was not correlated with plant diversity, density or biomass. Water infiltration rate was not correlated with leaching of the tested nutrients. Water infiltration or ammonium, nitrate and phosphate concentrations of leachates were not correlated with earthworm biomass (data not shown).

## Discussion

Our results show that the effects of earthworms on plant community density, diversity and production are altered by the presence of AM fungi. Of the two functional groups of earthworms, anecics and endogeics, that differ in their burrowing activity and feeding behaviour anecics appeared to be altered more by AMF than the endogeic species. Moreover, AMF root colonization differed between plant functional groups and was specifically affected by earthworm species. Water infiltration and nutrient leaching was directly affected only by earthworms, however indirect effects of AMF on earthworm activity also altered these important ecosystem functions via changes on plant community density, diversity and biomass.

AMF colonization levels of plant species were rather low but can be attributed to (i) the fact that AMF was inoculated only in a bottom layer of the mesocosms in about 20 cm distance from seeds and (ii) to the short duration of the experiment particularly focussing on the establishment phase of grasslands. Nevertheless, root AMF colonization of forbs and legumes was increased by the activity of *L. terrestris* indicating that this vertically burrowing species enabled the establishment of the symbiosis between plants and the distantly located AM fungi more than the more horizontally burrowing *A. caliginosa*. In grasses, however, the endogeic *A. caliginosa* stimulated AMF colonization more than *L. terrestris* possibly by creating a more beneficial burrowing system for the homogeneous grass root system that facilitated AMF symbiosis. The few studies focussing on earthworm-AMF interactions showed heterogeneous results encompassing reduced [66], increased [67] or no effects [53], [57], [56] of earthworms on AMF colonization.

### Effects on plant community characteristics

While the time of seedling emergence was unaffected by earthworms or AMF, *L. terrestris* significantly reduced the total number of emerging seedlings only when AMF was present while mixed earthworm communities or the endogeics alone did not influence seedling emergence. This has to our knowledge not been observed before and suggests that the sapro-geophageous anecic species was more stimulated by AMF to feed and remove seeds from the soil surface than the geophageous endogeic species.

AMF-stimulation of the anecic species also led to a 19% reduced plant community diversity while the earthworm mix or only endogeic earthworms showed no effect. While several studies showed effects of plant diversity on earthworms [6], [7], [68], [69] this is to the best of our knowledge the first one showing combined effects of both earthworms and AMF on plant community diversity. However, it has to be noted that the current study only focussed on the establishment phase of these communities and it has to be investigated whether these effects remain during further community development.

Density of plant communities decreased from 55 plants mesocosm^−1^ without earthworms to 37 plants mesocosm^−1^ in mixed earthworm treatments. Both the highest (NoEw) and the lowest plant densities (Lt and AcLt) were seen in the AMF inoculated mesocosms, however this pattern was unrelated to earthworm biomass as Lt treatments had a higher biomass than mixed earthworm treatments. Mesocosms without AMF showed decreased plant density in the earthworm mix due to a reduction of forbs, however when AMF were present, detrimental effects of the earthworm mix and anecics on plant density were more pronounced and affected both forb and legume densities. This again indicates that mainly anecic rather than endogeic earthworms have been stimulated by AMF, perhaps by providing food more utilisable by anecic than endogeic earthworms [47], [70]. Our current finding that earthworm effects vary with plant functional groups has frequently been reported [9], [21], [23], [24], [25], [71], [72]. However, it is difficult to draw some general conclusions from these studies as depending on the earthworm and/or plant species studied different response patterns were observed.

Contrasting to plant density and diversity, plant community biomass was only influenced by earthworms when AMF was absent. The highest shoot mass in Ac and Lt treatments and the lowest shoot mass in mixed earthworm treatments indicate that competition between earthworms in mixed communities decreased their effects on plant production. In mesocosms without AMF, endogeic and anecic earthworms had similar stimulating effects on shoot biomass production despite the fact that root production was higher when anecic Lt but not endogeic Ac were active, indicating great plasticity in biomass allocation of these communities. In mesocosms with AMF, earthworms did not influence total biomass production suggesting that differences in earthworm burrowing activities were levelled out by AMF. Although it has been shown that plant growth is specifically influenced by earthworm activity [73], further studies are needed to elucidate the underlying mechanisms. Perhaps the earthworm-AMF interaction complex is also responsible for often observed lack of earthworms on growth, especially in field studies where AMF is naturally present in the soil [23], [74].

### Effects on water infiltration and nutrient leaching

Several studies showed that the soil physical and chemical properties may vary, depending on differences in the burrowing system of earthworm species [31], [32], [33]. In our experiment water infiltration was significantly increased by earthworms confirming numerous findings in the literature that earthworm burrows functioned as preferential flow paths [75], [35], [36]. It was unexpected to see that also endogeic earthworms that are known to form more horizontal channels [76] increased infiltration rates. Similar findings were reported by [61] who attributed this to a higher burrowing activity and a higher number of connections between burrows of endogeic earthworms, relative to anecic species. When monitoring earthworm activity in the current experiment (data not shown) we also observed that our endogeic species created burrows with opening at the soil surface that indeed could facilitate water infiltration into the soil body. No correlation between earthworm biomass and water infiltration could be found in the current study, while in arable land dominated by anecics, water infiltration rates were correlated to earthworm biomass, burrow length, surface and volume [77]. We did not expect AMF to directly affect water infiltration rates especially in such a short-term study, however AMF effects might occur in the long-term via stimulated root growth or higher aggregate stability.

Both anecic and mixed anecic-endogeic earthworm treatments increased ammonium leaching indicating the higher mobility of ammonium-N than nitrate present in earthworm casts deposited by these species in the soil and at the soil surface [78]. In arable systems earthworms have been shown to increase nitrate leaching [37], indicating that under the less fertile conditions of the current experiment plant nitrate uptake was more readily leaving less nitrate in the soil for leaching. In contrast to recent studies [45], [46], phosphate leaching was unaffected by AMF, however this can be explained by the short duration of the current study, different AMF taxa and plant species investigated and the low root AMF colonisation. Another very important aspect of the current study was that effects of earthworms or AMF on plant community parameters indirectly induced water infiltration as well as ammonium and nitrate leaching, e.g. mesocosms containing 12 plant species showed twice as high nitrate concentrations than those with 15 plant species. Similar relationships between plant community diversity, density and biomass and nutrient leaching have been reported earlier [79], [80], however again, the particular assembly of plant species seems to trigger this relationship more than the plant species diversity *per se* [81], [82].

### Conclusions

To our knowledge, for the first time the results of this study demonstrate earthworm-AMF interactions with consequences on the diversity, structure and functioning of model grassland communities. For most of the tested parameters interactions between the sapro-geophageous anecic earthworms and AMF seem to be more prevalent than between the geophageous endogeics and AMF. Although these patterns were investigated in mesocosms only, our multi-species approach provides some clues how belowground-aboveground linkages [83] might work in more natural communities. Clearly, in order to better understand the underlying mechanisms and disentangle these complex interactions more experiments are necessary using earthworm communities comprising anecics, endogeics and epigeics and more diverse AMF communities. Nevertheless, given the decisive role that earthworms and AM fungi play in grasslands the implications of our findings regarding other climate changes besides a projected increase in heavy rainfalls can be appreciated [21], [23], [84], [85].
